# Supplementary material for: New Small-Molecule SERCA Inhibitors Enhance Treatment Efficacy in Lenvatinib-Resistant Papillary Thyroid Cancer
Source: Int J Mol Sci. 2024 Oct 3;25(19):10646. doi: 10.3390/ijms251910646 (PMC11476702; doi:10.3390/ijms251910646)
Supplement: Supplementary file 1 [file ijms-25-10646-s001.zip › ijms-3100359-supplementary.pptx]

## Slide 1
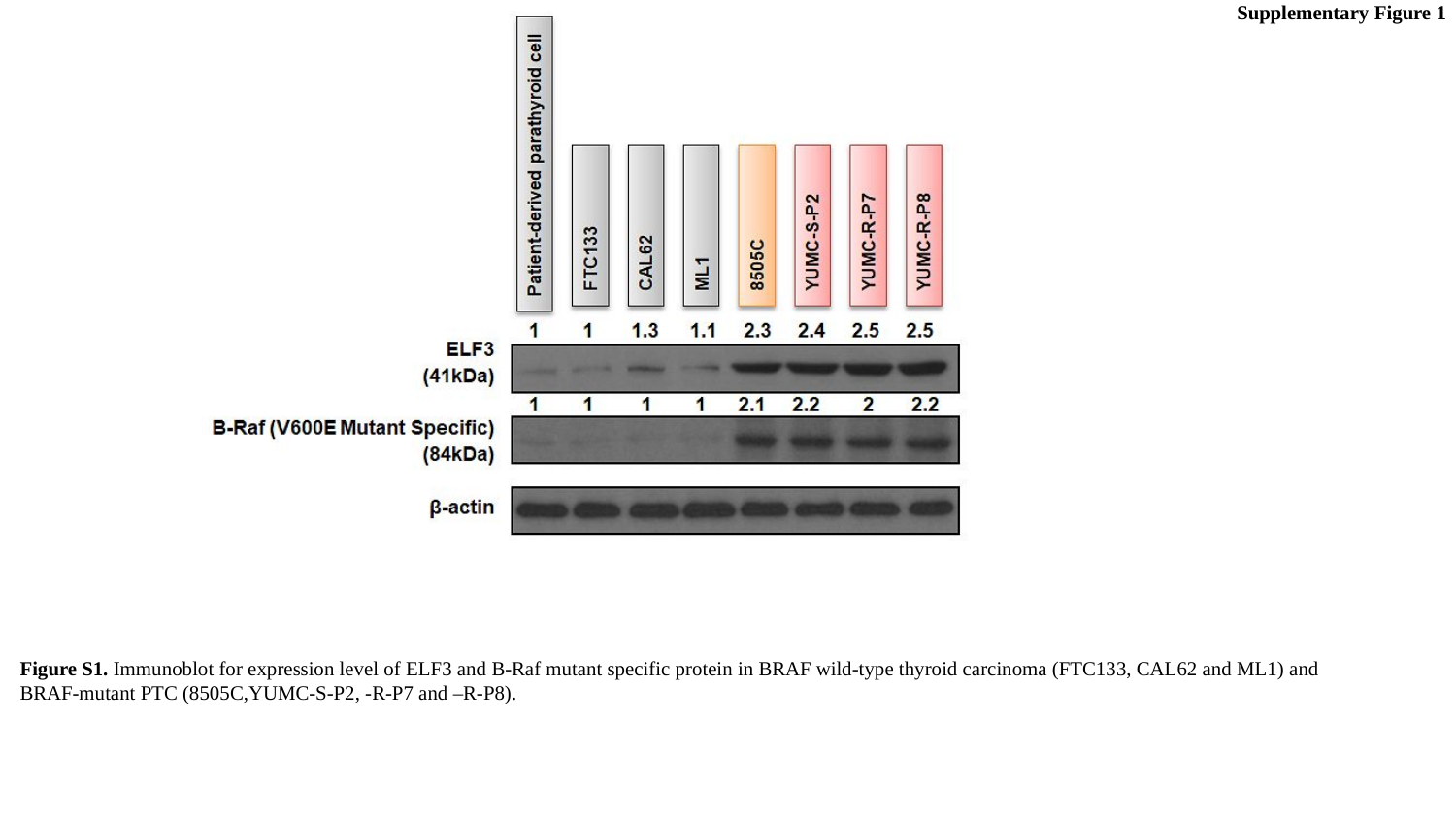

Supplementary Figure 1
Figure S1. Immunoblot for expression level of ELF3 and B-Raf mutant specific protein in BRAF wild‑type thyroid carcinoma (FTC133, CAL62 and ML1) and BRAF‑mutant PTC (8505C,YUMC-S-P2, -R-P7 and –R-P8).

## Slide 2
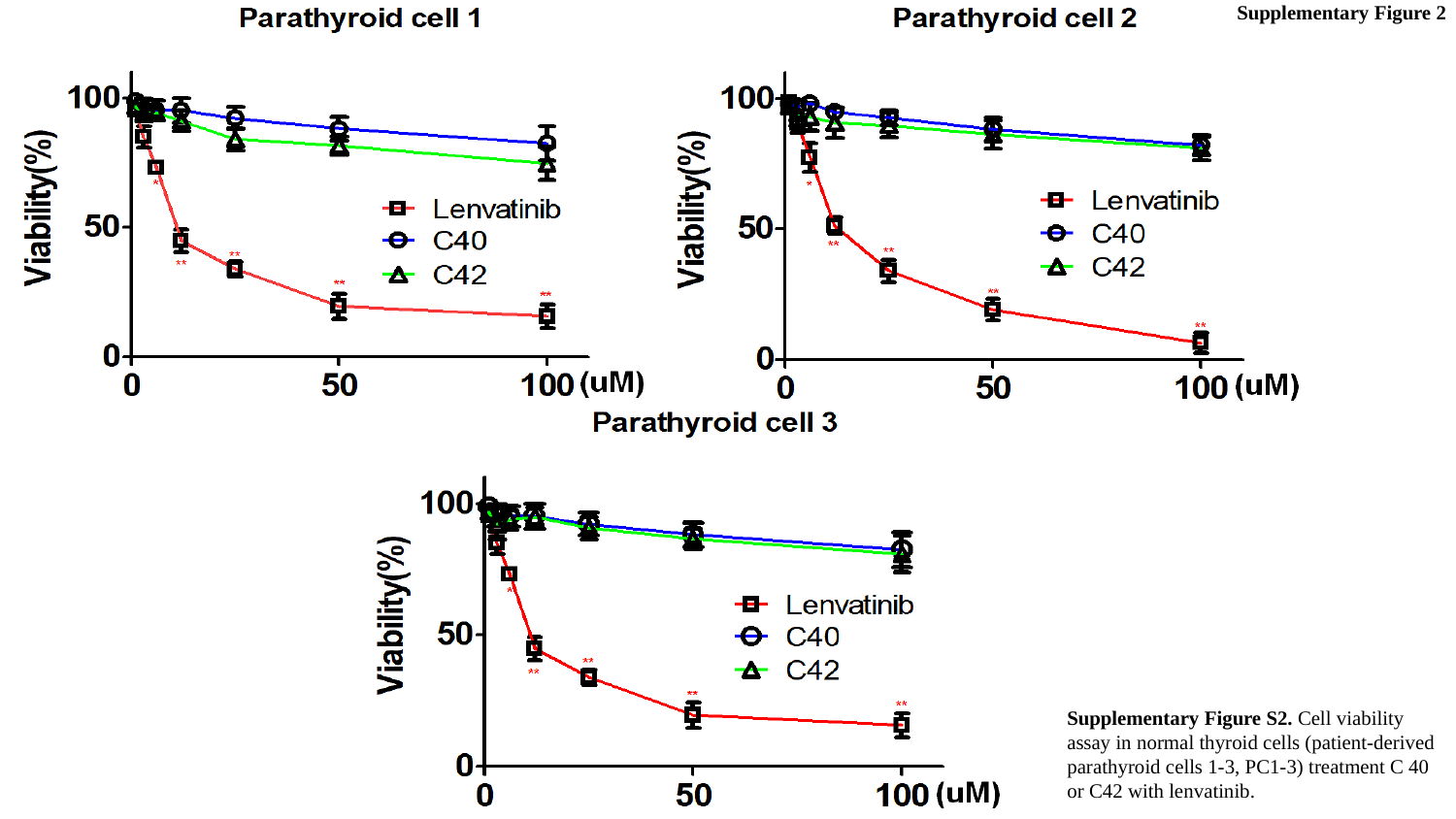

Supplementary Figure 2
Supplementary Figure S2. Cell viability assay in normal thyroid cells (patient-derived parathyroid cells 1-3, PC1-3) treatment C 40 or C42 with lenvatinib.
